# RB in DNA repair

**DOI:** 10.18632/oncotarget.5234

**Published:** 2015-08-22

**Authors:** Paul H. Huang, Rebecca Cook, Sibylle Mittnacht

**Affiliations:** The Institute of Cancer Research, Division of Cancer Biology, Chester Beatty Laboratories and CancerCell Signalling, UCL Cancer Institute, University College London, London, UK

**Keywords:** retinoblastoma protein, DNA repair

The retinoblastoma protein (RB1) has a welldocumented role as a key regulator of cell cycle progression by controlling the G1/S phase transition [1]. RB1 has also emerged as a multi-functional protein involved in a wide range of biological processes including transcriptional regulation by recruiting chromatin remodelling enzymes, DNA replication via interaction with DNA polymerase complex components and apoptosis through association with the mitochondria [1]. In our recently published study [2], we add to this functional repertoire by demonstrating that RB1 and its paralogs p107 and p130 play a central role in DNA double strand break (DSB) repair by non-homologous end joining (NHEJ).

By employing an affinity purification proteomics strategy, our study finds that RB1, through its amino terminal (RB1^N^) domain, binds to components of the NHEJ machinery including Ku70, Ku80 and DNAdependent protein kinase (DNA-PK). We further show using structure-guided mutations that these interactions are dependent on a conserved cyclin wedge homology surface within RB1^N^. Importantly, engineered RB1 mutants disabled for Ku70 binding were unable to rescue NHEJdependent DNA repair when expressed in RB1-negative cells. Consistent with these data, cells with RB1 loss displayed increased frequency of chromosomal aberrations upon irradiation which is a hallmark of defective NHEJ. A key finding of our study is that the capacity of RB1 to regulate NHEJ is genetically separate from its canonical functions in cell cycle progression or E2F transcriptional regulation.

Our study adds to an increasing body of evidence that RB1 is important for maintaining genomic stability in response to overt DNA damage. RB1 depletion leads to an increase in chromosome instability (CIN), manifesting in aneuploidy or polyploidy [3]. Widespread chromosome gains and loss associated with RB1 loss is attributed to centromere dysfunction and the failure to recruit components of the Condensin II complex, leading to a defect in chromosome condensation during mitosis [3]. RB1 also regulates global chromatin structure and consequently gene expression through the recruitment of key chromatin modifying enzymes. These include histone deacetylases HDAC 1 and 2, histone methyltransferase SUV4 and SWI/SNF chromatin remodelling complex catalytic subunit Brahman/SWI2-related gene (BRG1), all of which have been shown to be important for DNA DSB repair [1, 4]. Furthermore, RB1 binds to tumour protein p53 binding protein 1 (53BP1) via a methylated K810 residue which directly links RB1 function to the DNA damage response [5]. Together these data argue that RB1 is a key player in preventing genome instability through a complex interplay of regulatory events including centromere function, chromatin structure and direct recruitment by DNA damage repair proteins.

Other than the well-established context in cancer and the role of RB1 as a bona fide tumour suppressor, RB1 may also be important in DNA damage surveillance during aging. Human cells are naturally subjected to DNA damage insults, such as oxidative stress from metabolic processes, which if left unrepaired would lead to accumulation of damage within both the nuclear and mitochondrial DNA. Amitotic cells such as skeletal muscle cells or neurons are particularly susceptible to accumulation of DNA damage over time and it is thought that this is likely to be a prominent cause of aging. Consistent with this idea, mice with defective mutations in the NHEJ proteins Ku70 and Ku80 display a premature aging phenotype without increased cancer incidence levels [6]. Gene deletion in mice has provided indirect evidence that RB1 is necessary for maintaining survival of fully differentiated post-mitotic neurons in mice [7]. Acute loss of RB1 in neurons induces the expression of cell cycle proteins with a corresponding increase in DNA double strand breaks, leading to cell death in vivo. A tempting hypothesis is that RB1 is part of the NHEJ surveillance machinery in the face of naturally occurring DNA damage in amitotic cells and may be a key player for preventing age-associated neurodegenerative disorders.

There remain many open questions as to the role of RB1 in facilitating NHEJ. These include establishing if RB1 is itself recruited to sites of DNA DSBs, the mechanism(s) by which RB1 assembles DNA repair proteins and accessory factors at DSBs, and if there is a direct link between RB1 function in NHEJ and its ability to regulate chromatin structure through modifiers such as HDAC 1 and 2. Future work in elucidating these mechanisms will enable the exploitation of this novel function of RB1 for new therapies in cancer and ageassociated disorders
